# The Role of E3 Ubiquitin Ligase Cbl Proteins in β-Elemene Reversing Multi-Drug Resistance of Human Gastric Adenocarcinoma Cells

**DOI:** 10.3390/ijms140510075

**Published:** 2013-05-10

**Authors:** Ye Zhang, Xiao-Dong Mu, En-Zhe Li, Ying Luo, Na Song, Xiu-Juan Qu, Xue-Jun Hu, Yun-Peng Liu

**Affiliations:** 1 Department of Medical Oncology, the First Hospital of China Medical University, Shenyang 110001, China; E-Mails: zhangye228@hotmail.com (Y.Z.); medical_oncology@yahoo.cn (E.-Z.L.); luoying_cmu@hotmail.com (Y.L.); sgna1980@yahoo.com.cn (N.S.); xiujuanqu@yahoo.com (X.-J.Q.); 2 Department of Respiratory Medicine, the First Hospital of China Medical University, Shenyang 110001, China; E-Mail: medical_oncology1@yahoo.cn

**Keywords:** E3 ubiquitin ligase, β-elemene, multidrug resistance, PI3K/Akt

## Abstract

Recent studies indicate that β-elemene, a compound isolated from the Chinese herbal medicine Curcuma wenyujin, is capable of reversing tumor MDR, although the mechanism remains elusive. In this study, β-Elemene treatment markedly increased the intracellular accumulation of doxorubicin (DOX) and rhodamine 123 in both K562/DNR and SGC7901/ADR cells and significantly inhibited the expression of P-gp. Treatment of SGC7901/ADR cells with β-elemene led to downregulation of Akt phosphorylation and significant upregulation of the E3 ubiquitin ligases, c-Cbl and Cbl-b. Importantly, β-elemene significantly enhanced the anti-tumor activity of DOX in nude mice bearing SGC7901/ADR xenografts. Taken together, our results suggest that β-elemene may target P-gp-overexpressing leukemia and gastric cancer cells to enhance the efficacy of DOX treatment.

## 1. Introduction

Patients undergoing chemotherapy for treatment of various cancers often develop resistance to a wide variety of anti-cancer drugs, a phenomenon known as multidrug resistance (MDR). Overexpression of p-glycoprotein (P-gp), an integral membrane protein, represents one of the major mechanisms contributing to the MDR phenotype [1]. It is believed that inhibition of P-gp function or inhibition of its expression may reverse P-gp-mediated MDR and improve the effectiveness of chemotherapy. Since the early 1980s, a broad spectrum of compounds has been examined for their capability to overcome P-gp-mediated MDR. Notable examples include verapamil, phenothiazines, and cyclosporins. Unfortunately, despite their potent anti-P-gp activity in cultured cells, the clinical trials of these compounds have not yet been successful, in large part because of their confounded pharmacokinetic interaction with anticancer drugs and their notorious side effects. Numerous studies have confirmed that overexpression of P-gp in tumor cells correlates with poor prognosis following chemotherapy [2–5]. Therefore, inhibition of P-gp expression may reverse the MDR phenotype by enhancing intracellular accumulation of anti-cancer drugs.

Elemene, extracted from the Chinese herbal medicine Curcuma wenyujin, is a mixture of β-, γ- and δ-elemene. β-Elemene, the main active component, has been shown to exhibit anti-proliferative effects in various types of cancer, including leukemia [6], prostate [7], lung [8,9] and ovarian cancer [10]. Inhibition of cell proliferation and induction of apoptosis have been proposed as the primary mechanisms underlying the anti-tumor effects of β-elemene [11–14]. Recently, β-elemene was approved as a national first-class new agent and phase II clinical trials are currently underway [15]. Several studies have shown that this novel agent enhances sensitivity to chemotherapy in human cancer cell lines. One study demonstrated that treatment of a chemoresistant human breast cancer cell line (MCF-7/D) with β-elemene led to reversion of MDR [16]. However, the ability of β-elemene to enhance the efficacy of DOX in leukemia and gastric cancer cells remains unclear.

In neoplastic cells, phosphatidylinositol-3 kinase (PI3K) signaling has been shown to control cell growth through activation of downstream pathways mediated by the serine/threonine protein kinase, Akt. Inhibition of PI3K/Akt signaling can lead to decreased cell growth and tumor formation [17]. Previously, we showed that inhibition of the PI3K/Akt pathway significantly reverses MDR in leukemia and gastric cancer [18]. Other studies also reported that LY294002, a specific inhibitor of the PI3K/Akt kinase pathway, antagonizes P-gp-mediated MDR in the L1210/VCR murine leukemic cell line [19]. In addition, McCubrey *et al*., reported that activated forms of Akt can increase drug resistance of prostate cancer cells [20,21]. Furthermore, studies by Choi *et al*., demonstrated that inhibition of PI3K/Akt signaling down-regulates P-gp expression [22]. Taken together, these results suggest that regulation of the PI3K/Akt pathway might have profound effects on P-gp-mediated MDR.

The Casitas B-lineage lymphoma (Cbl) family of ubiquitin ligases are negative regulators of non-receptor tyrosine kinases and some activated signaling pathways [23]. The TKB domain of the Cbl family of proteins can interact with the p85 subunit of PI3K, leading to ubiquitination and degradation [24,25]. Our group also reported that Cbl-b overexpression strongly inhibits Akt phosphorylation in human gastric cancer cells [26]. Collectively, these data suggest that Cbl family proteins (c-Cbl and Cbl-b) may be involved in regulating sensitivity to anti-cancer drugs by modulating PI3K/Akt signaling. Here, we explore the mechanisms underlying the ability of β-elemene to enhance doxorubicin (DOX) chemosensitivity in leukemia and gastric cancer cells. We demonstrate that by up-regulating the expression of c-Cbl and Cbl-b, which leads to inhibition of PI3K/Akt signaling and down-regulation of P-gp expression, β-elemene is capable of enhancing the efficacy of DOX in leukemia and gastric cancer cells.

## 2. Results and Discussion

### 2.1. *In Vitro* Cytotoxicity of β-Elemene

**Figure 1:**
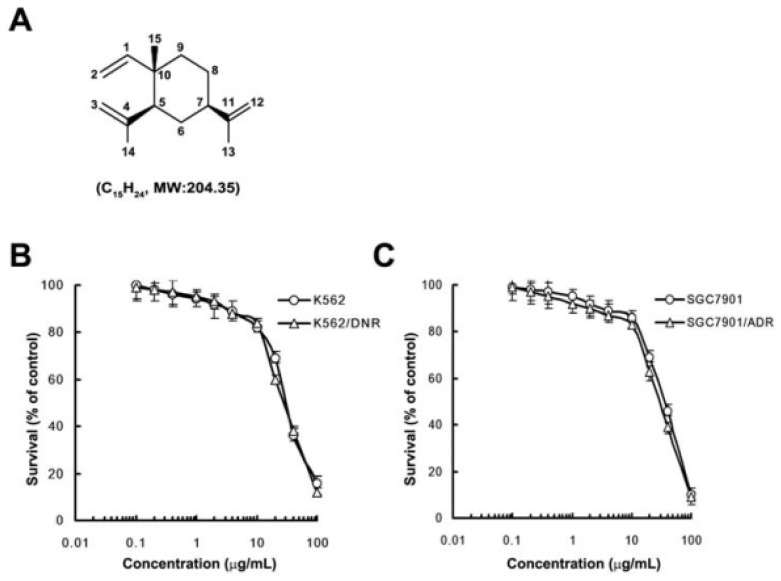
Structure and cytotoxic effects of β-elemene. (**A**) The structure of β-elemene; (**B**) Cell viability in K562 and P-gp-overexpressing K562/DNR cells was assessed by trypan blue exclusion assay; (**C**) Cell viability in SGC7901 and P-gp-overexpressing SGC7901/ADR cells was assessed by MTT assay. Cells were exposed to increasing concentrations of β-elemene for 48 h. Each point represents the mean ± standard deviation (SD) for three determinations. Each experiment was performed in three replicate wells.

We first examined the cytotoxic effects of β-elemene on K562 leukemia and SGC7901 gastric adenocarcinoma cell lines and their drug-resistant derivatives (K562/DNR and SGC7901/ADR, respectively) with overexpression of P-gp, by trypan blue exclusion assay or MTT assay. No significant difference in the cytotoxicity of β-elemene was observed between the parental and resistant cells (Figure 1, IC_50_ 18.66 ± 1.03, 16.31 ± 0.67, 35.05 ± 1.99, 34.42 ± 2.91 μg/mL for K562, K562/DNR, SGC7901, SGC7901/ADR cells, respectively). More than 90% of cells were viable following treatment with 10 μg/mL β-elemene; therefore, concentrations of 1, 5 and 10 μg/mL were used in the MDR reversal study.

### 2.2. β-Elemene Reverses P-gp-Mediated MDR *in Vitro*

The IC_50_ values of chemotherapeutic agents in MDR cells and their parental drug-sensitive derivatives treated either alone or in combination with various concentrations of β-elemene are shown in Table 1. Verapamil, a specific inhibitor of P-gp, was used as a positive control. The overexpression of P-gp was associated with significantly higher IC_50_ values of substrate drugs [18,26]. β-Elemene strongly reduced the IC_50_ values of conventional chemotherapeutic agents in K562/DNR and SGC7901/ADR cells. Treatment with β-elemene (10 μg/mL) led to a 16.55-, 14.57-, and 15.13-fold reversal of resistance to DOX, DNR and EPI in K562/DNR cells and a 8.01-, 11.02- and 10.87-fold reversal to these drugs in SGC7901/ADR cells. However, the sensitivity to these chemotherapeutic agents was not altered when parental K562 and SGC7901 cells were treated concomitantly with β-elemene. Furthermore, β-elemene did not alter the IC_50_ value of non-P-gp substrates (cisplatin) in either MDR or sensitive cells.

### 2.3. β-Elemene Reverses P-gp-Mediated MDR *in Vivo*

**Figure 2:**
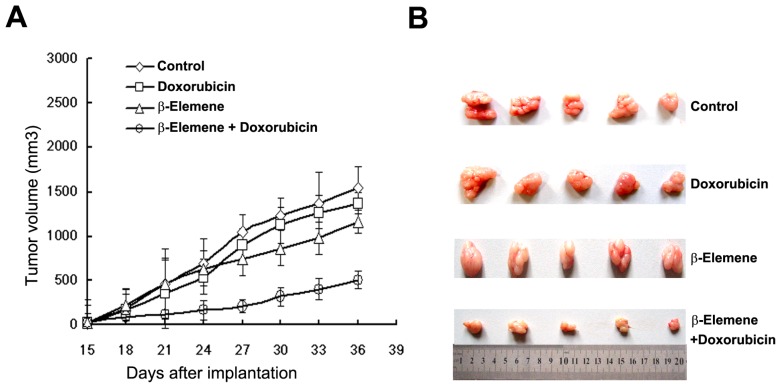
Potentiation of anti-tumor effects of DOX by β-elemene in a SGC7901/ADR xenograft model in nude mice. (**A**) Changes in tumor volume with time. Each point represents the mean ± SD of tumor volumes from five mice in the group. Mice were treated with saline (control, ⋄); β-elemene alone at 25 mg/kg (□); DOX alone at 2 mg/kg (Δ); DOX at 2 mg/kg plus β-elemene 25 mg/kg (○) (β-elemene was given 1 h before DOX administration); (**B**) Tumor size. The image was taken on the 36th day post-implantation.

We next tested the ability of β-elemene to reverse drug resistance to DOX *in vivo* using an established SGC7901/ADR xenograft model in nude mice. As shown in Figure 2, neither DOX nor β-elemene significantly inhibited the growth of tumor xenografts. However, the combination of DOX and β-elemene drastically inhibited the growth of SGC7901/ADR xenografts in nude mice (tumor weight 1.92 ± 0.26 g, 1.79 ± 0.37 g, 1.83 ± 0.53 g and 0.82 ± 0.22 g for saline, DOX, β-elemene and combination groups, respectively). The inhibition rate of the combination group was 42.6%. We did not observe significant body weight loss or treatment-related deaths in the combination group, indicating that β-elemene effectively enhanced the anti-tumor activity of DOX without causing additional toxicity.

### 2.4. β-Elemene Increases the Accumulation of Rho 123 and DOX in Both K562/DNR and SGC7901/ADR Cells

**Figure 3:**
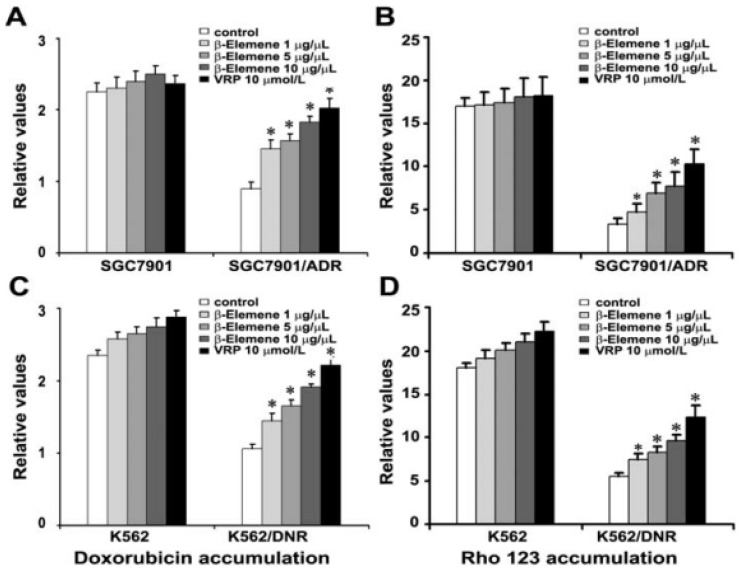
Effect of β-elemene on the accumulation of doxorubicin and rhodamine 123. (**A**–**D**) The accumulation of doxorubicin was measured by flow cytometric analysis as described in Section 2. The results are presented as fold change in fluorescence intensity relative to control MDR cells. Fold change was calculated by dividing the fluorescence intensity of each sample with that of MDR cells treated with doxorubicin or rhodamine 123 alone. Columns represent the mean of triplicate determinations ± SD. * *p* < 0.05 *versus* control group.

Intracellular Rho 123-associated MFI was employed to study the effects of β-elemene on inhibition of P-gp function. DOX is also a good substance for ABC transporters, and agents that inhibit transporter function are capable of increasing the accumulation of DOX in drug-resistant cells. As shown in Figure 3, treatment of both K562/DNR and SGC7901/ADR cells with β-elemene (1, 5 and 10 μg/mL) led to a significant increase in Rho 123 and DOX MFI compared with control treated cells (*p* < 0.05). However, the intracellular accumulation of Rho 123 and DOX was not altered in SGC7901 cells in the presence of β-elemene. These results suggest that β-elemene is capable of inhibiting the drug transport activity of P-gp in both K562/DNR and SGC7901/ADR cells.

### 2.5. Effect of β-Elemene on the Inhibition of Akt Phosphorylation and Expression of P-gp

**Figure 4:**
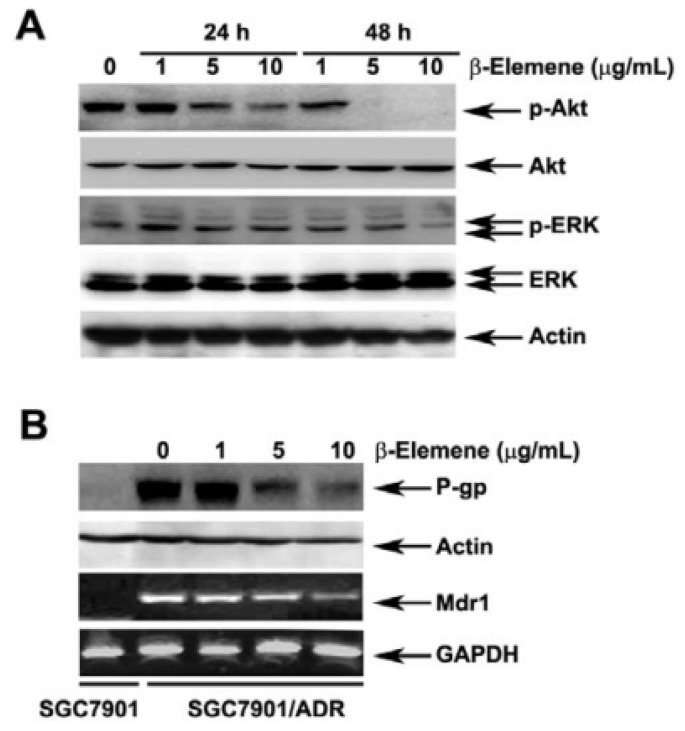
Effect of β-elemene on inhibition of Akt and ERK1/2 phosphorylation and the expression of P-gp in MDR cells. (**A**) SGC7901/ADR cells were treated with β-elemene at 1, 5 and 10 μg/mL for 24 h and 48 h. Equal amounts of protein were analyzed by western blot as described in Section 2; (**B**) SGC7901/ADR cells were treated with β-elemene at 1, 5 and 10 μg/mL for 48 h. The expression of P-gp at both protein and mRNA levels was analyzed as described in Section 2. SGC7901 cells were used as negative controls. Independent experiments were performed at least three times, and a representative experiment is shown.

To understand whether the inhibition of PI3K/Akt and/or MAPK/ERK pathways underlies the reversal of MDR mediated by β-elemene, we next examined the phosphorylation of Akt and ERK1/2 in SGC7901/ADR cells. As shown in Figure 4A, treatment of cells with β-elemene (1, 5 and 10 μg/mL) for 24 h led to a significant decrease in phosphorylated Akt but not ERK1/2.

The reversal of ABC transporter-mediated MDR is typically obtained by down-regulating the expression of transporters. Therefore, we investigated the effect of β-elemene on the expression of P-gp at the mRNA and protein levels. Our results showed that the P-gp protein and mRNA expression were down-regulated following treatment with β-elemene at the indicated concentrations (Figure 4B).

### 2.6. Effect of β-Elemene on the Up-Regulation of c-Cbl and Cbl-b Expression

**Figure 5:**
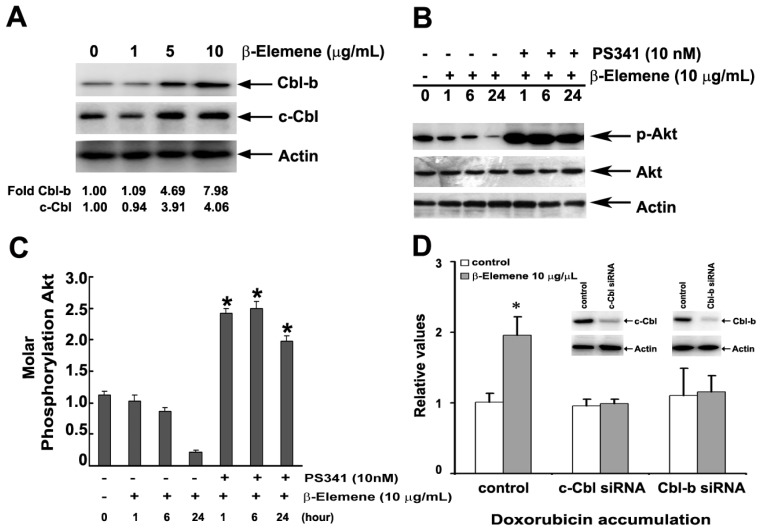
Effect of β-elemene on c-Cbl and Cbl-b expression in MDR cells. (**A**) SGC7901/ADR cells were treated with β-elemene at 1, 5 and 10 μg/mL for 24 h. The expression of c-Cbl and Cbl-b was detected as described in Section 2; (**B**) After exposure to 10 μg/mL β-elemene, the expression of p-Akt, Akt and β-Actin was measured by western blot in the presence or absence of 10 nM PS341; (**C**) The expression of p-Akt following β-elemene and/or PS341 treatment in experiment B was estimated using NIH Image software. The mean ± S.E. (*n* = 3) from three separate experiments are presented. * *p* < 0.05 *versus* control group; (**D**) SGC7901/ADR cells were transiently transfected with c-Cbl and Cbl-b specific siRNA for 48 h, followed by 10 μg/mL β-elemene for 24 h, and the intracellular accumulation of DOX was measured by flow cytometric analysis as described in Section 2. Columns represent the mean of triplicate determinations ± SD. * *p* < 0.05 *versus* control group.

To investigate whether c-Cbl and Cbl-b ubiquitin ligases play a role in the reversal of MDR mediated by β-elemene, we examined the expression of these proteins following treatment. As shown in Figure 5A, c-Cbl and Cbl-b proteins were significantly up-regulated in SGC7901/ADR cells following treatment with 5 and 10 μg/mL of β-elemene for 24 h. These results indicate that Cbl proteins are involved in the reversal of MDR mediated by β-elemene, and possibly lie upstream of the PI3K/Akt signaling pathways. Then, SGC7901/ADR cells were pre-treated for 30 min with PS341, a proteasome inhibitor that suppresses the functions of Cbl proteins, followed by incubation with β-elemene. The expression of p-Akt in cells pre-treated with PS341 was higher than in untreated control cells at 1 h, 6 h and 24 h, suggesting that Cbl proteins are involved in the reversal of MDR mediated by β-elemene (Figure 5B,C). To confirm the role of Cbl proteins in MDR, we used RNA interference technique to knockdown the endogenous c-Cbl and Cbl-b proteins expression in human gastric adenocarcinoma SGC7901/ADR cells. We found that β-elemene does not reverse MDR in SGC7901/ADR cells that transiently transfected specific c-Cbl and Cbl-b siRNAs (Figure 5D).

### 2.7. Discussion

β-Elemene is a new anti-tumor compound with potent anti-growth and anti-proliferative activities in a broad range of cancer cell types, including lung cancer, ovarian cancer, breast cancer and prostate cancer [11,14,27,28]. In China, β-elemene has been used to effectively treat certain types of tumors in the clinic, and it presents fewer side effects than other cytotoxic agents [29,30]. Recently, β-elemene has been studied as an agent capable of reversing resistance to chemotherapy. Xu *et al*., reported the reversion of MDR in a chemoresistant human breast cancer cell line by β-elemene [16]. Studies by Li *et al*., confirmed enhancement of cisplatin-induced apoptosis by β-elemene in resistant human ovarian cancer cells [31]. Previously, we demonstrated the synergistic anti-tumor effect of β-elemene and etoposide in non-small cell lung carcinoma cells [32]. However, whether β-elemene may be used in combination with conventional P-gp substrate chemotherapeutic drugs to overcome MDR in leukemia and gastric cancer cells remains unclear.

In the present study, we demonstrate that β-elemene significantly enhanced the cytotoxicity of established P-gp substrates including DOX, DNR and EPI, in MDR leukemia and gastric cancer cells. In contrast, the cytotoxicity generated by the P-gp substrates was unaffected in the presence of β-elemene in drug-sensitive cells. K562/DNR and SGC7901/ADR cells are drug resistance models with expression of P-gp [18,26]. Treatment of cells with β-elemene (10 μg/mL) led to the significant reversal of resistance to DOX, DNR and EPI in both K562/DNR and SGC7901/ADR cells (Table 1). This suggests that β-elemene may represent a promising compound for use in combination with chemotherapeutic drugs. Furthermore, β-elemene did not significantly alter the sensitivity of non-P-gp substrates such as cisplatin, in sensitive and resistant cells. In athymic nude mice bearing SGC7901/ADR xenografts, β-elemene drastically enhanced the anti-tumor activity of DOX without causing additional toxicity (Figure 2). Our data also revealed that β-elemene was capable of enhancing the chemotherapeutic sensitivity of DOX by increasing the accumulation of DOX and Rho 123 in SGC7901/ADR and K562/DNR cells (Figure 3A–D).

The reversal of ABC transporter-mediated MDR can usually be obtained by down-regulating the expression of transporters. In the present study, we examined the effect of β-elemene on the expression of P-gp mRNA and protein. We found that P-gp protein and mRNA expression was down-regulated following β-elemene treatment. Indeed, several studies have demonstrated that inhibition of Akt activation can overcome MDR in leukemia and in MDR prostate cancer cell lines [10,23,24]. In the present study, we observed a significant inhibitory effect on the phosphorylation levels of Akt in cells treated with different concentrations of β-elemene for 24 h. These results indicate that β-elemene reverses P-gp-mediated MDR via inhibition of PI3K/Akt signaling.

Regulation of the PI3K/Akt signaling pathway is complex. Recent studies suggest that the ubiquitin ligase Cbl-b, which ubiquitinates important signaling molecules, may play a central role in down-regulating PI3K [26,33]. The p85 regulatory subunit of PI3K has been identified as a substrate for Cbl-b, and Cbl proteins direct PI3K ubiquitination. Previously, we showed that increased expression of Cbl-b can circumvent P-gp-mediated MDR in gastric cancer cells by suppression of PI3K/Akt signaling and down-regulation of P-gp expression [26]. To investigate whether ubiquitin ligases c-Cbl and Cbl-b are involved in the reversal of MDR mediated by β-elemene, the expression of these proteins was examined in SGC7901/ADR and K562/DNR cells after treatment with β-elemene. As shown in Figure 5, c-Cbl and Cbl-b were significantly up-regulated in SGC7901/ADR and K562/DNR cells, indicating that these proteins are involved in the reversal of P-gp-mediated MDR by β-elemene. Pre-treatment of SGC7901/ADR cells with PS341 (a suppressor of Cbl proteins), followed by incubation with β-elemene led to upregulation of p-Akt at 1 h, 6 h and 24 h compared with control pre-treated cells. Further studies investigating the biological function and regulation of c-Cbl and Cbl-b are required in order to evaluate the possibility that agents intervening in this pathway may be of clinical utility in modulating the MDR phenotype during cancer chemotherapy.

## 3. Experimental Section

### 3.1. Reagents

β-Elemene was obtained from Jingang Pharmaceutical (Dalian, China). Antibodies against p-ERK, ERK, c-Cbl, Cbl-b, P-gp and β-actin were purchased from Santa Cruz Biotechnology Inc. (Santa Cruz, CA, USA). Antibodies specific to Akt and phospho-Akt (p-Akt) were purchased from Cell Signaling Technology (Beverly, MA, USA). Rhodamine 123 (Rho 123), 1-(4,5-demethylthiazol-2-yl)-3,5-diphenyltetrazoliumbromide (MTT), DOX, daunorubicin (DNR), epirubicin (EPI), verapamil and other chemicals were obtained from Sigma Chemical Co. (St. Louis, MO, USA).

### 3.2. Cell Lines and Culture

The K562 human leukemia cell line and its DNR-selected P-gp overexpressing derivative cell line, K562/DNR, were kindly provided by Professor T. Ueda (Fukui Medical University, Japan) [18]. The SGC7901 human gastric adenocarcinoma cell line was preserved in our institution and its DOX-selected P-gp overexpressing derivative cell line, SGC7901/ADR, was granted by the Institute of Gastroenterology, Xijing Hospital, Fourth Military Medical University [26]. All cell lines were cultured in RPMI 1640 supplemented with 10% fetal bovine serum at 37 °C in the presence of 5% CO_2_.

### 3.3. Cell Viability Assay

The effect of β-elemene on K562 and K562/DNR cell proliferation was assessed by trypan blue exclusion assay. K562 and K562/DNR cells were collected by centrifugation and mixed with an equal volume of PBS containing 0.4% trypan blue dye, and cell number was determined using a hemocytometer and taking the dilution factor in account. Cell viability (%) was calculated as follows:

(1) $$\text{Cell viability }(\%)=\frac{\text{viable cell numbers}}{\text{total }(\text{viable}+\text{dead})\;\text{cell numbers}}\times 100\%$$

The effect of β-elemene on SGC7901 and SGC7901/ADR cell proliferation was assessed by MTT assay as previously described [34]. Briefly, SGC7901 and SGC7901/ADR cells (3 × 10^4^/well) were seeded in 96-well plates and incubated for 24 h. Cells were treated with various concentrations of β-elemene for 48 h, and 25 μL of MTT solution (5 mg/mL) was added to each well and incubated for 4 h at 37 °C. Following removal of culture medium, cells were lysed in 200 μL of DMSO and the O.D. was measured at 570 nm using a microplate reader (Model 550, Bio-Rad Laboratories, Hercules, CA, USA). The degree of resistance was calculated by dividing the IC_50_ for MDR cells by that of the parental sensitive cells. The fold-reversal factor of MDR was calculated by dividing the IC_50_ following treatment with chemotherapeutic drugs in the absence of β-elemene by that obtained in the presence of β-elemene.

### 3.4. Animal and Tumor Xenograft Experiments

Male athymic nude mice (BALB/c-nu/nu) (4–6 weeks, 18–24 g), were purchased from Shanghai Slike Experimental Animals Co. (animal experimental license no. SCXKhu2007-0005, Shanghai, China) and housed under specific, pathogen-free conditions. Approximately 5 × 10^6^ SGC7901/ADR cells were injected subcutaneously into the posterior flanks region of nude mice. Mice bearing tumors ≈100 mm^3^ in volume (two weeks after tumor inoculation) were randomized into five mice per group. A total of four groups of mice bearing SGC7901/ADR xenografts were treated with: (a) phosphate-buffered saline (q3d × 7, i.p); (b) β-elemene (25 mg/kg, q3d × 7, i.p.); (c) DOX (2 mg/kg, q3d × 7, i.p.); (d) DOX (2 mg/kg, q3d × 7, i.p.) plus β-elemene (25 mg/kg, q3d × 7, i.p.). Animal body weight and two perpendicular diameters (*A* and *B*) were recorded every 3 days. Tumor volume (*V*) was calculated as:

(2) $$V=\frac{\pi }{6}{\left(\frac{A+B}{2}\right)}^{3}$$

Mice were sacrificed when the average tumor weight was over 1 g in the control group, and tumor tissue was excised from mice and weighed. The rate of inhibition (*IR*) was calculated as:

(3) $$IR(\%)=1-\frac{\text{Mean tumor weight of experimental group}}{\text{Mean tumor weight of control group}}\times 100\%$$

### 3.5. DOX and Rho 123 Accumulation

The effect of β-elemene on the intracellular accumulation of DOX and Rho 123 in SGC7901 and SGC7901/ADR cell lines was measured by flow cytometry as previously described [26]. Briefly, SGC7901 and SGC7901/ADR cells (3 × 10^5^ cells/well) were incubated in six-well plates overnight. Cells were treated with 1, 5 and 10 μg/mL β-elemene at 37 °C for 3 h. DOX (10 μmol/L) or Rho 123 (5 μg/mL) was then added and cells were incubated for 2 h. The intracellular mean fluorescence intensity (MFI) associated with DOX and Rho 123 was determined by FACScan flow cytometry (Becton Dickinson, San Jose, CA, USA). A minimum of 10,000 cells were analyzed for each histogram generated.

### 3.6. Small Interfering RNA Transfections

Cells were seeded at a density of 3 × 10^5^ cells/well in 6-well plates. After 24 h, cells were transfected with small interfering RNA (siRNA) using Lipofectamine 2000 reagent according to the manufacturer’s protocols. The c-Cbl siRNA sequence used was: CUG CCG AUG UGA AAU UAA ATT. The Cbl-b siRNA sequence used was: AAA GTG GTA AGA CTG TGC CAA. The Ctrl sequence used was: AAT TCT CCG AAC GTG TCA CGT. c-Cbl, Cbl-b and Ctrl siRNAs were prodeced from Genechem Co. (Shanghai, China). Gene silencing effect was verified by Immunoblotting.

### 3.7. Western Blot Analysis

Cells were extracted and protein was quantified as described previously [26]. Aliquots (50 μg) of each lysate were separated by electrophoresis on SDS-PAGE gels and transferred to nitrocellulose membranes. Membranes were blocked with 5% non-fat milk in TBST (10 mM Tris-HCl pH 7.4, 100 mM NaCl, 0.5% Tween-20) for 2 h at room temperature and incubated overnight at 4 °C in 5% non-fat milk in TBST containing P-gp, p-Akt, Akt, p-ERK, ERK, c-Cbl, Cbl-b or β-actin antibodies. Membranes were washed and incubated with peroxidase-conjugated second antibodies for 1 h. After extensive washing with TBST, proteins were visualized using the enhanced chemiluminescence reagent (SuperSignal Western Pico Chemiluminescent Substrate; Pierce, Rockford, IL, USA). Final images were analyzed using NIH Image J software.

### 3.8. Reverse-Transcription Polymerase Chain Reaction (RT-PCR)

Cells were treated with β-elemene for 48 h, collected by centrifugation and washed twice with ice-cold PBS. Total RNA was extracted using the RNeasy mini kit (Qiagen, Carlsbad, CA, USA) as described by the manufacturer. Reverse transcription was performed as follows: 1 μg of RNA was combined with 1 μL of random hexamer primers (0.5 μg/μL) and incubated for 10 min at 70 °C. First strand buffer (4 μL), 0.1 M dithiothreitol (2 μL), dNTP mix (1 μL) and Superscript II (1 μL) (Invitrogen, Carlsbad, CA, USA) were then added to each sample and incubated for 1 h at 42 °C and 15 min at 70 °C. PCR was performed using 50 ng of cDNA, and *P-gp* gene expression was determined as described [33].

### 3.9. Statistical Analysis

Data are presented as the mean ± standard deviation, and differences were determined using the Student’s *t*-test. A *p* value of less than 0.05 was considered statistically significant. All means were calculated from at least three independent experiments.

## 4. Conclusions

β-elemene enhances the efficacy of conventional chemotherapeutic drugs in P-gp-overexpressing leukemia and gastric cancer cells by downregulating the expression of P-gp and increasing the intracellular concentrations of substrate chemotherapeutic drugs. In addition, the phosphorylation of Akt was downregulated and the expression of E3 ubiquitin ligases c-Cbl and Cbl-b, were significantly upregulated in two P-gp-overexpressing cells following β-elemene treatment. Our results suggest that β-elemene may be used in combination with conventional P-gp substrate chemotherapeutic drugs to overcome MDR in the clinic.

## Figures and Tables

**Table 1:** Effect of β-elemene on reversing P-gp-mediated drug resistance.

Group	IC_50_ ± SDs μmol/L (fold reversal)		IC_50_ ± SDs μmol/L (fold reversal)	
		
	K562	K562/DNR	SGC7901	SGC7901/ADR
Doxorubicin	0.096 ± 0.012 (1.00)	5.014 ± 0.106 (1.00)	2.032 ± 0.041 (1.00)	20.011 ± 0.006 (1.00)
+1 μg/mL β-elemene	0.086 ± 0.009 (1.12)	2.219 ± 0.092 ** (2.26)	2.026 ± 0.011 (1.01)	14.276 ± 0.092 ** (1.40)
+5 μg/mL β-elemene	0.088 ± 0.017 (1.09)	0.519 ± 0.036 ** (9.65)	2.014 ± 0.016 (1.01)	4.003 ± 0.107 ** (5.00)
+10 μg/mL β-elemene	0.082 ± 0.022 (1.17)	0.303 ± 0.024 ** (16.55)	2.002 ± 0.022 (1.02)	2.503 ± 0.012 ** (8.01)
+10 μM verapamil	0.081 ± 0.034 (1.18)	0.231 ± 0.017 ** (21.67)	2.002 ± 0.024 (1.02)	2.202 ± 0.106 ** (9.09)
Daunorubicin	0.0091 ± 0.0004 (1.00)	1.229 ± 0.021 (1.00)	0.288 ± 0.011 (1.00)	2.269 ± 0.013 (1.00)
+1 μg/mL β-elemene	0.0088 ± 0.0005 (1.03)	1.538 ± 0.009 ** (3.26)	0.279 ± 0.007 (1.03)	1.881 ± 0.007 ** (1.21)
+5 μg/mL β-elemene	0.0090 ± 0.0003 (1.01)	0.707 ± 0.012 ** (7.09)	0.280 ± 0.013 (1.03)	0.764 ± 0.090 ** (2.97)
+10 μg/mL β-elemene	0.0090 ± 0.0002 (1.01)	0.344 ± 0.032 ** (14.57)	0.281 ± 0.022 (1.02)	0.206 ± 0.022 ** (11.02)
+10 μM verapamil	0.0087 ± 0.0001 (1.04)	0.238 ± 0.025 ** (20.09)	0.277 ± 0.014 (1.04)	0.129 ± 0.025 ** (17.59)
Epirubicin	0.019 ± 0.005 (1.00)	0.963 ± 0.009 (1.00)	2.114 ± 0.013 (1.00)	18.075 ± 0.061 (1.00)
+1 μg/mL β-elemene	0.018 ± 0.003 (1.04)	0.231 ± 0.004 ** (4.16)	2.069 ± 0.009 (1.02)	11.296 ± 0.004 ** (1.60)
+5 μg/mL β-elemene	0.019 ± 0.003 (1.02)	0.133 ± 0.007 ** (7.24)	2.046 ± 0.010 (1.03)	5.124 ± 0.013 ** (3.53)
+10 μg/mL β-elemene	0.017 ± 0.012 (1.07)	0.064 ± 0.002 ** (15.13)	2.043 ± 0.032 (1.03)	1.663 ± 0.067 ** (10.87)
+10 μM verapamil	0.018 ± 0.003 (1.05)	0.043 ± 0.005 ** (22.53)	2.046 ± 0.019 (1.03)	0.993 ± 0.025 ** (18.20)
Cisplatin	3.174 ± 0.012 (1.00)	2.861 ± 0.036 (1.00)	3.991 ± 0.040 (1.00)	4.203 ± 0.109 (1.00)
+10 μg/mL β-elemene	2.784 ± 0.097 (1.14)	2.983 ± 0.103 (0.96)	3.905 ± 0.011 (1.02)	4.119 ± 0.078 (1.02)
+10 μM verapamil	2.994 ± 0.021 (1.06)	2.778 ± 0.015 (1.03)	3.823 ± 0.013 (1.04)	4.108 ± 0.062 (1.02)

Cell survival was determined by trypan blue exclusion assay or MTT assay as described in Materials and Methods, IC_50_ values were calculated by nonlinear regression analysis using GraphPad Prism version 5.00 for Windows (GraphPad Software, San Diego, CA, USA). Data represent the mean ± standard deviation (SD) of at least three independent experiments performed in triplicate. The fold reversal of MDR was calculated by dividing the IC_50_ of the chemotherapeutic drugs in the absence of β-elemene or inhibitor by that obtained in the presence of β-elemene or inhibitor.

** *p* < 0.01 *versus* that obtained in the absence of inhibitor.
